# Immediate Effects of Dry Needling in Patients with Myofascial Temporomandibular Disorders: A Randomized Controlled Trial

**DOI:** 10.3390/healthcare14142189

**Published:** 2026-07-20

**Authors:** Nurcan Durmaz, Serdar Gözler, Gamze Demircioğlu, Kevser Burma

**Affiliations:** 1 Department of Prosthodontics, Faculty of Dentistry, Istanbul Atlas University, 34408 İstanbul, Türkiye; nurcan.durmaz@atlas.edu.tr; 2 Department of Physiotherapy and Rehabilitation, Faculty of Health Sciences, Istanbul Atlas University, 34408 İstanbul, Türkiye; gamze.demircioglu@atlas.edu.tr (G.D.); kevser.burma@atlas.edu.tr (K.B.)

**Keywords:** temporomandibular disorders, myofascial pain, dry needling, electromyography, trigger points, orofacial pain

## Abstract

**Background**: Myofascial temporomandibular disorders (TMDs) are associated with pain and trigger points in the masticatory muscles. Dry needling (DN) is used for myofascial pain, but evidence regarding immediate effects remains limited. **Objective**: This participant- and outcome-assessor-blinded randomized controlled trial evaluated immediate clinical and electromyographic effects of a single DN session targeting the masseter and anterior temporalis muscles in patients with myofascial TMD. **Methods**: Forty-four participants diagnosed according to the Research Diagnostic Criteria for Temporomandibular Disorders were randomly allocated to a DN group or a sham needling group. Pain intensity was assessed using a visual analog scale, and surface electromyographic activity of the masseter and anterior temporalis muscles was recorded at rest and during maximal clenching. Measurements were obtained before treatment and 10 min after the intervention. Adverse events were recorded throughout the observation period. **Results**: Forty participants completed the study, with 20 in each group. Pain intensity decreased significantly in the DN group compared with the sham group (*p* < 0.001). An uncorrected analysis indicated reduced resting activity of the left anterior temporalis muscle; however, no electromyographic variable remained significant after correction for multiple comparisons. No serious adverse events occurred. Minor transient bleeding and mild post-needling soreness were reported in a small number of participants receiving DN. **Conclusions**: A single DN session produced a significant immediate reduction in pain intensity in patients with myofascial TMD, without consistent immediate changes in surface electromyographic activity. Longer follow-up and repeated-session studies are needed to determine the durability and clinical relevance of these effects.

## 1. Introduction

Temporomandibular disorders (TMDs) comprise a broad spectrum of conditions involving the temporomandibular joint, masticatory musculature, and related craniofacial structures, frequently presenting with pain, functional restriction, and compromised mandibular performance. Rather than representing a single disease entity, TMD encompasses multiple musculoskeletal and neuromuscular disorders with diverse etiologies and clinical manifestations [1]. Population-based evidence indicates that temporomandibular disorders affect a substantial proportion of the general population, with recent meta-analytic estimates suggesting a global prevalence of approximately 34%. TMDs are reported more frequently among women, with adults aged 18–60 years representing the most affected age group [2].

Among the diagnostic categories of TMD, myofascial pain represents one of the most common muscle-related disorders. The DC/TMD classification distinguishes local myalgia from myofascial pain with referral according to the distribution of pain elicited during clinical examination, thereby improving diagnostic precision for muscle-related TMD conditions [3]. These conditions are frequently associated with muscular tenderness, functional limitation, and reduced quality of life [4,5]. Myofascial TMD is commonly characterized by localized or referred pain arising from hypersensitive trigger points located within taut bands of skeletal muscle [6]. These trigger points are believed to contribute to abnormal sensory input, altered muscle coordination, and persistent nociceptive activity, thereby playing an important role in symptom maintenance.

Current understanding suggests that myofascial TMD develops through an interaction of peripheral biomechanical factors and central pain-processing mechanisms. Excessive muscular loading, parafunctional habits, psychosocial stressors, and maladaptive neuromuscular responses may all contribute to the development or persistence of symptoms [2,7,8]. Because of this multifactorial pathophysiology, conservative multimodal approaches are generally recommended as the primary management strategy. The evolution of TMD diagnostic concepts has been described previously [9].

Common first-line interventions include patient education, physiotherapy, behavioral modification, pharmacologic approaches, and occlusal management strategies designed to reduce pain and improve mandibular function [4,7]. Within this therapeutic spectrum, dry needling (DN) has emerged as a minimally invasive technique increasingly used for the management of myofascial trigger points.

Dry needling involves insertion of a thin filiform needle directly into trigger points or dysfunctional muscle tissue with the objective of reducing pain and restoring neuromuscular balance [10]. Proposed mechanisms include local mechanical disruption of trigger point activity, normalization of dysfunctional endplate responses, enhancement of local blood flow, and modulation of both peripheral and central nociceptive pathways [10,11,12]. In addition to localized physiological effects, DN may also influence broader pain regulatory systems through activation of endogenous inhibitory mechanisms.

Previous studies have reported favorable effects of dry needling in patients with myofascial pain, including reduced pain intensity, improved jaw mobility, and functional improvement in TMD-related symptoms [13,14,15,16,17,18]. However, although the existing literature generally supports short-term therapeutic benefit, most prior investigations have focused on outcomes assessed after multiple sessions or across follow-up periods extending from days to weeks. Consequently, the immediate post-treatment effects of a single DN intervention—particularly within minutes of application—remain insufficiently characterized.

This distinction is clinically relevant because understanding whether dry needling can induce measurable immediate analgesic or neuromuscular changes may clarify its early mechanisms of action and help determine whether DN may serve not only as a cumulative therapeutic modality but also as an acute adjunctive intervention for rapid symptom modulation.

Therefore, the present participant- and outcome-assessor-blinded randomized controlled trial was designed to investigate the immediate effects of a single session of dry needling applied to the masseter and anterior temporalis muscles in individuals diagnosed with myofascial temporomandibular disorders. Specifically, this study aimed to compare immediate changes in subjective pain intensity and objective surface electromyographic activity between dry needling and sham intervention conditions. We hypothesized that dry needling would produce superior immediate pain reduction compared with sham treatment while potentially influencing early neuromuscular responses.

## 2. Materials and Methods

### 2.1. Study Design and Participants

This study, conducted between 28 July and 8 August 2025, was designed as a participant- and outcome-assessor-blinded randomized controlled trial at the Faculty of Dentistry at Istanbul Atlas University. Ethical approval was obtained from the Institutional Review Board and Ethics Committee of Istanbul Atlas University, in accordance with the Declaration of Helsinki (ethical board approval code: E-22686390-050.99-72665 and study clinical registration number (CRN) is NCT07123883). Before commencement of the study, eligible participants were informed of the study purpose and procedures, assured that they could withdraw at any time, and provided written consent.

Patients aged 18–45 years who presented with complaints of jaw pain to the Faculty of Dentistry at Atlas University were considered eligible for this study. At the time of clinical assessment, all participants were diagnosed with myofascial pain associated with temporomandibular dysfunction using the Axis I criteria of the Research Diagnostic Criteria for Temporomandibular Disorders (RDC/TMD) [19]. The inclusion criteria included the ability to achieve an interincisal opening between 30 and 50 mm without restriction [20]; jaw pain persisting for at least three months; a pain intensity of 40 mm or higher on the 100 mm Visual Analog Scale (VAS) [21]; pain elicited upon palpation of the masseter and/or temporalis muscles; presence of clicking or crepitus in the temporomandibular joint during mandibular movements; and self-reported jaw muscle fatigue or stiffness, particularly upon awakening. At the time of protocol initiation, RDC/TMD criteria were used for participant classification to maintain methodological consistency, while contemporary DC/TMD literature informed the conceptual framework of the study.

The exclusion criteria were a confirmed or suspected inflammatory or neurological condition; an oral or dental infection; a cognitive or communication impairment affecting data collection; a history of head or facial trauma; a metal allergy; a circulatory disorder requiring anticoagulant or related medication; use of psychotropic, narcotic, corticosteroid, analgesic, or anti-inflammatory medication; botulinum toxin injections within the previous six months; or any other jaw, facial, or temporomandibular-region treatment that could influence pain perception or muscle activity during the study period.

### 2.2. Sample Size Calculation

The sample size calculation was based on the Masseter sEMG values reported for temporomandibular joint pain by Blasco-Bonora and Martín-Pintado-Zugasti [22]. Using an effect size (Cohen’s d) of 1.02, a significance level (α) of 0.05, and a statistical power (1 − β) of 0.85, a two-tailed independent sample *t*-test indicated that 36 participants (18 per group) were required to detect significant differences between the groups. To account for potential dropouts, the total sample size was increased by 20%, resulting in a planned enrollment of 44 participants (22 per group).

### 2.3. Study Protocol

Participants were allocated to either the dry needling group or the sham needling group using simple randomization generated with the random number generator available at Random.org. Allocation concealment was maintained using sealed, opaque envelopes prepared by an independent researcher who was not involved in intervention delivery or outcome assessment. Owing to the nature of the procedures, the physiotherapist administering the intervention was aware of group allocation, whereas the participants and the outcome assessor remained blinded throughout the study. The sham procedure mimicked the sensation of needle insertion without penetrating muscle tissue to maintain participant blinding. The study comprised pre-intervention, intervention, and post-intervention stages.

### 2.4. Pre-İntervention Assessment

All evaluations were conducted after instructing participants to refrain from engaging in strenuous physical activity on the day of assessment. Only individuals experiencing jaw pain on either the right or the left side were considered eligible for inclusion. Following the collection of medical and dental history, each participant underwent a comprehensive physical examination.

Jaw pain intensity was evaluated at the end of the range of motion using a 100 mm VAS, a tool recognized for its high reliability in musculoskeletal assessments. All participants were asked to indicate their pain level on the VAS, allowing for subjective quantification of perceived pain intensity [23].

**Figure 1:**
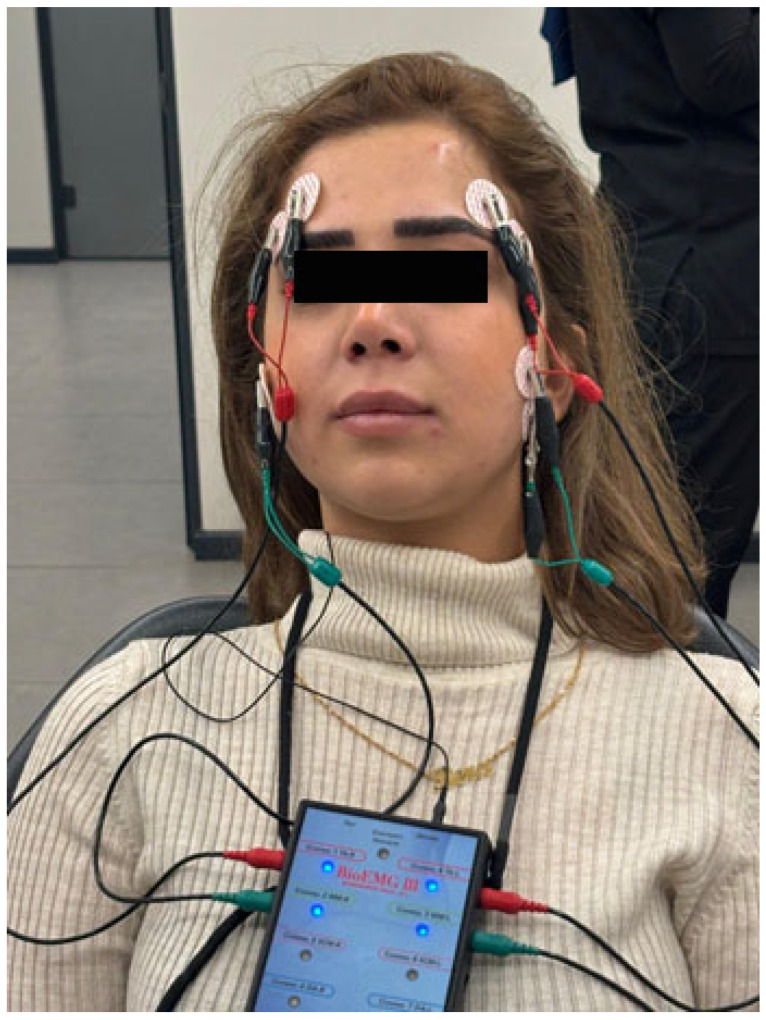
The surface electromyographic (sEMG) activity of the jaw muscles was recorded bilaterally from the anterior temporalis (TA-R, TA-L) and masseter (MM-R, MM-L) muscles.

Surface electromyographic (sEMG) activity of the jaw muscles was recorded bilaterally from the anterior temporalis (TA-R, TA-L) and masseter (MM-R, MM-L) muscles using the BioPAK System (version 7.2; BioResearch Associates Inc., Milwaukee, WI, USA) (Figure 1). The BioEMG III unit is an eight-channel electromyography system designed to record craniofacial muscle activity at rest and during function, with data acquisition and visualization integrated through BioPAK software [24].

Recordings were obtained under two standardized conditions: (1) resting position, with the mandible relaxed, lips gently closed, and teeth not in contact; and (2) maximal voluntary clenching, performed in the intercuspal position for 5 s with verbal encouragement. The sEMG recordings were conducted under standardized laboratory conditions using the BioPAK System (BioResearch Associates Inc., Milwaukee, WI, USA). Prior to electrode placement, the participants were instructed to avoid wearing facial makeup or applying any topical substances, and to shave the skin area if necessary. The skin overlying the target muscles was cleansed thoroughly with 95% isopropyl alcohol and allowed to air-dry.

Resting EMG Recording: Surface electrodes were carefully placed bilaterally over the anterior temporalis and masseter muscles with a ground electrode attached according to the manufacturer’s guidelines. Once the amplifier box indicator showed a green light (indicating a proper connection), all EMG presets were adjusted to 30 s, except for the resting protocol. The room lighting was dimmed, and the participants were instructed to sit upright with both feet flat on the floor, close their eyes, and remain relaxed without engaging their masticatory muscles. The Resting EMG Preset was selected to initiate automatic recording. At the midpoint of the 30 s trace, the recording was briefly paused, and participants were instructed to stand up and then resume the resting posture to complete the recording.

Clenching EMG Recording: Participants were instructed to perform three maximal voluntary clenches (approximately 2 s each) on their posterior natural dentition. The recording was then paused, and standard cotton rolls were placed between the maxillary and mandibular anterior teeth. Three additional clenches were performed under these conditions. Subsequently, the procedure was repeated with cotton rolls repositioned on the posterior teeth.

For each condition, three trials were recorded with a 1 min rest interval, and the mean values were used for the analysis. The sEMG signals were acquired using BioFLEX surface electrodes (BioResearch Associates Inc., Milwaukee, WI, USA) consisting of two rectangular conductive polyester adhesive contacts (surface area, 144 mm^2^; inter-electrode distance, 20 mm). The skin was cleaned with 95% alcohol prior to electrode placement, which followed established surface EMG recommendations and masticatory muscle-specific guidance [25,26]. Raw sEMG data were initially stored in the TRC format and converted to ASCII (.txt) files using the BioPAK software and subsequently imported into MATLAB (version R2017a; MathWorks Inc., Natick, MA, USA) for processing with custom-written scripts. From artifact-free recordings, root mean square (RMS) amplitudes were calculated and expressed in microvolts (µV), with mean and standard deviation (SD) values reported.

### 2.5. Interventions

All participants were randomly assigned to one of two groups: the dry needling group (DN group) or sham needling group (SG). In the DN group, deep dry needling was administered to the most sensitive myofascial trigger point identified in either the masseter or anterior temporalis muscle using a sterile acupuncture needle (0.20 × 13 mm, Qinzhou, China). The procedure was performed by a physiotherapist with seven years of clinical experience under the supervision of a licensed physician.

**Figure 2:**
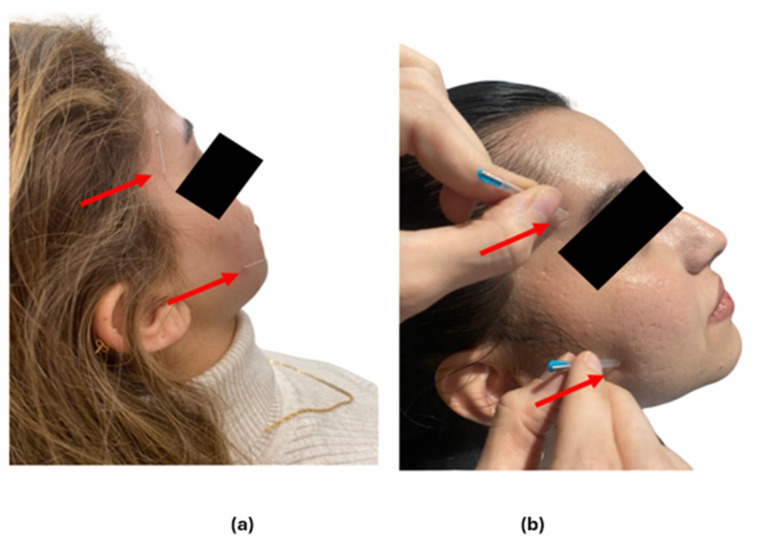
Dry needling intervention: (**a**) Dry needling procedure; (**b**) Sham needling procedure.

Trigger points were identified by palpation of the masseter and temporalis muscles by the principal investigator and were marked on the skin using a white dermatographic pencil. Prior to intervention, the marked area was disinfected with alcohol. Participants were placed in a supine position with their eyes closed. To ensure optimal access, the head was rotated to the contralateral side during needling (i.e., to the right for left-side treatment and vice versa) in accordance with standard protocols reported in previous studies [27,28] (Figure 2). In the sham needling group, the same anatomical regions were approached, but the needle was applied superficially to mimic the sensory experience of needling without penetrating the muscle tissue. No intramuscular depth was reached and no local twitch response was elicited.

### 2.6. Post-Intervention Assessment

After the intervention, participants rested for a standardized 10 min period to allow the initial neuromuscular responses to stabilize before post-treatment measurements. All outcome measures were then reassessed using the same procedures as at baseline.

To maintain objectivity, all measurements were conducted by a secondary evaluator who was blinded to group allocation and treatment procedures. Pain intensity was reassessed using the VAS by the same blinded evaluator. Apart from the physiotherapist who administered the dry needling, both the participants and evaluators were blinded to the intervention groups throughout the study.

The occurrence of any unintended reactions or treatment-related events was assessed immediately after the intervention and throughout the subsequent observation period by the blinded evaluator.

### 2.7. Statistical Analysis

Statistical analyses were performed using IBM SPSS Statistics Version 26. The normality of the distribution of demographics and clinical characteristics of all participants in the participant groups was assessed using the Kolmogorov–Smirnov test. Between-group comparisons were conducted using the independent *t*-test for parametric data and Mann–Whitney U test for non-parametric data. Within-group comparisons of pre- and post-treatment data were performed using the paired *t*-test for parametric variables and the Wilcoxon signed-rank test for non-parametric variables. Differences were considered statistically significant at *p* < 0.05. To reduce the risk of type I error associated with multiple EMG comparisons, the Benjamini–Hochberg false discovery rate (FDR) correction procedure was additionally applied.

Analyses were performed on participants who completed both baseline and post-intervention assessments (per-protocol analysis).

## 3. Results

**Figure 3:**
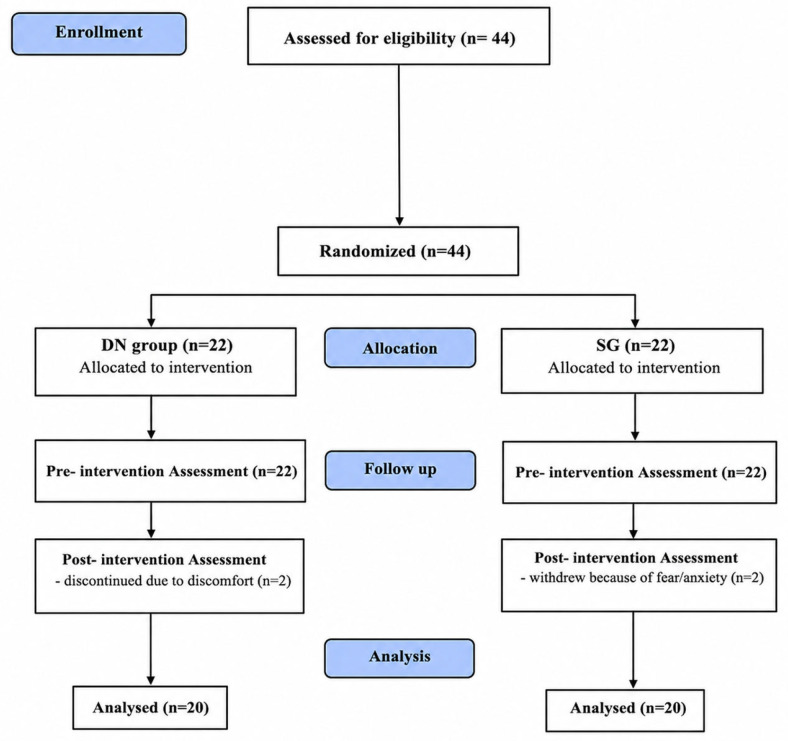
CONSORT flow diagram illustrating participant recruitment, randomization, follow-up, and final analysis.

The study was conducted and reported in accordance with the CONSORT guidelines for randomized controlled trials. A total of 44 participants meeting the inclusion criteria were initially enrolled and randomly allocated to either the dry needling group (DN group, n = 22) or the sham needling group (SG, n = 22). During the intervention period, four participants (two from each group) withdrew from the study. In the DN group, two participants discontinued participation because of discomfort associated with the dry needling procedure, whereas in the SG, two participants withdrew because of fear or anxiety related to the intervention. Consequently, 40 participants (20 per group) completed the study and were included in the final analysis. The participant recruitment and allocation process is illustrated in Figure 3.

The demographic and baseline clinical characteristics of the participants are presented in Table 1. The two groups were comparable in terms of age and baseline outcome measures, and no statistically significant differences were observed between the groups at baseline (*p* > 0.05). The mean age of the participants was 26.90 ± 8.92 years in the DN group and 27.70 ± 6.35 years in the SG.

The within-group and between-group comparisons of pre- and post-intervention outcomes are summarized in Table 2.

Within-group analysis revealed a statistically significant reduction in pain intensity measured by the visual analog scale (VAS) in the DN group following the intervention (*p* < 0.001). In contrast, no statistically significant change in VAS scores was observed in the SG (*p* > 0.05).

With respect to electromyographic outcomes, no significant changes were observed in the sEMG activity of the masseter or anterior temporalis muscles at rest or during maximal voluntary clenching within either group (*p* > 0.05).

Between-group comparisons demonstrated a statistically significant reduction in VAS scores in favor of the DN group compared with the SG (*p* < 0.001). An initial analysis suggested a decrease in the resting sEMG activity of the left anterior temporalis (TA-L) muscle in the DN group compared with the SG (*p* = 0.04). However, this difference did not remain statistically significant after correction for multiple comparisons.

No serious adverse events were reported during the study. Minor transient bleeding and mild post-needling soreness were observed in a small number of participants in the DN group, and these effects resolved spontaneously without requiring additional treatment.

## 4. Discussion

The present participant- and outcome-assessor-blinded randomized controlled trial investigated whether a single session of dry needling could produce immediate measurable effects in patients with myofascial temporomandibular disorders. The principal finding was that dry needling resulted in a statistically significant immediate reduction in pain intensity within 10 min of intervention, whereas sham treatment did not produce a comparable effect. In contrast, objective electromyographic parameters of the anterior temporalis and masseter muscles did not demonstrate consistent significant alterations after correction for multiple comparisons.

Collectively, these findings suggest that the earliest therapeutic benefit of dry needling in myofascial TMD may be more strongly associated with acute pain modulation than with immediate detectable changes in global muscle activation. This immediate-response framework distinguishes the present study from most previous dry needling investigations, which have predominantly focused on short-term or medium-term outcomes.

To our knowledge, this study is among the few randomized controlled trials specifically designed to evaluate the ultra-short-term (<10 min) effects of dry needling on both subjective pain perception and surface electromyographic activity in patients with myofascial TMD. This distinction is important because previous investigations have largely concentrated on outcomes measured after repeated sessions or over short-term follow-up periods lasting several days or weeks [16,17,18]. By specifically focusing on the immediate post-intervention window, the present study provides additional insight into the potential early neurophysiological effects of dry needling.

The rapid pain reduction observed in the dry needling group is consistent with previous literature demonstrating that trigger point dry needling can decrease pain intensity in myofascial pain disorders [14,15,16,17,18]. Several mechanisms may explain this immediate analgesic response. Mechanical stimulation of trigger points may disrupt dysfunctional motor endplate activity and reduce localized nociceptive input [6,10]. In addition, needle insertion may stimulate A-delta fibers and activate spinal or trigeminal inhibitory interneurons, thereby enhancing descending pain inhibition [10,12]. Biochemical studies have also suggested that dry needling may alter the local inflammatory environment by reducing concentrations of pain-related mediators associated with active trigger points [12]. Although the exact contribution of each mechanism remains uncertain, the present findings support the concept that dry needling can exert clinically relevant analgesic effects rapidly after a single application.

Despite the reduction in pain, the absence of robust electromyographic changes raises important considerations. Surface electromyography reflects overall muscle electrical behavior; however, conventional amplitude-based parameters may not be sufficiently sensitive to capture subtle or highly localized neuromuscular alterations immediately after intervention, and complementary signal descriptors have been proposed for jaw muscle sEMG analysis [29]. It is plausible that pain reduction may precede measurable motor adaptations, particularly when treatment exposure is limited to a single session. Previous studies examining dry needling in masticatory muscles have similarly reported variable or inconsistent EMG findings despite symptomatic improvement [16]. Therefore, the current dissociation between pain and sEMG outcomes does not necessarily indicate lack of physiological effect, but rather may suggest that immediate analgesia occurs through nociceptive modulation before broader functional muscular reorganization becomes detectable.

This apparent discrepancy may also reflect the complexity of myofascial TMD itself. Pain perception in temporomandibular disorders is influenced not only by local muscular pathology but also by central sensitization, psychosocial influences, and altered trigeminal pain processing [2]. Consequently, interventions such as dry needling may produce early reductions in pain through modulation of sensory processing pathways without immediately changing gross muscle recruitment patterns. From a clinical perspective, this distinction is meaningful because even transient pain relief may improve patient tolerance for adjunctive therapies such as therapeutic exercise, splint therapy, or behavioral interventions.

Importantly, the magnitude of pain reduction observed in the dry needling group exceeded commonly recognized thresholds for minimal clinically important difference in musculoskeletal pain, suggesting that the observed effect was not only statistically significant but also clinically relevant. This finding may support the use of dry needling as an early-stage symptom-modulating strategy within broader conservative TMD management protocols.

Several limitations should be acknowledged. First, outcomes were evaluated only during the immediate 10 min post-treatment period, precluding conclusions regarding sustained or cumulative therapeutic benefit. Second, the intervention involved a single treatment session, whereas routine clinical protocols may involve repeated applications. Third, although subjective pain and sEMG were assessed, other clinically important domains, including jaw function, psychosocial status, disability-related outcomes, lateral mandibular movements, and movement patterns such as deviation and deflection, were not evaluated. Trigger-point identification was based on manual palpation and may therefore have been subject to examiner-dependent variability. Participant blinding effectiveness was not formally tested. In addition, temporomandibular joint disk position was not confirmed using imaging; consequently, potential intra-articular heterogeneity among participants cannot be excluded.

Future research should investigate repeated-session protocols, longer follow-up durations, and multidimensional outcome frameworks to better define both the short- and long-term role of dry needling in myofascial TMD management. Integration of advanced neurophysiological or imaging modalities may further clarify whether early analgesic effects are primarily mediated by peripheral trigger point disruption, central neuromodulation, or a combination of both mechanisms.

In summary, the present findings indicate that dry needling can provide immediate pain relief in myofascial TMD even when measurable changes in muscle electrical activity are limited. These results support the potential value of dry needling as a conservative adjunctive intervention while highlighting the need for further investigation of its mechanisms and durability.

## 5. Conclusions

This randomized controlled trial demonstrated that a single session of DN administered to the masseter and temporalis muscles resulted in a statistically significant immediate reduction in pain intensity in patients with myofascial TMD. The absence of consistent sEMG changes suggests that the early analgesic effects may be more closely related to neuromodulatory mechanisms than to immediate alterations in muscle activity. These findings support DN as a potentially useful conservative adjunct for immediate pain modulation in myofascial TMD. Future studies should evaluate repeated treatment sessions, longer follow-up periods, and broader outcome domains to clarify the therapeutic role and underlying mechanisms of DN.

## Figures and Tables

**Table 1:** Demographics and clinical characteristics of all participants.

Variable	DN Group Mean ± SD	SG Mean ± SD	*p*
Age (yr)	26.90 ± 8.92	27.70 ± 6.35	0.74
Weight (kg)	62.30 ± 9.21	61.85 ± 10.74	0.88
Height (cm)	168.80 ± 6.66	164.60 ± 6.37	0.61
BMI (kg/m^2^)	21.80 ± 2.33	22.79 ± 3.77	0.28
VAS activity (mm)	62.3 ± 17.1	62.5 ± 10.2	0.73

**Table 2:** Within-group and between-group comparisons of pre- and post-intervention outcomes.

Variable	DN Group				SG				Between- Group *p*
	Pre Mean ± SD	Post Mean ± SD	Δ Mean ± SD	*p*	Pre Mean ± SD	Post Mean ± SD	Δ Mean ± SD	*p*	
VAS activity (mm)	62.3 ± 17.1	49.5 ± 10.9	13.0 ± 11.7	<0.001	62.5 ± 10.2	61.0 ± 10.2	1.5 ± 7.4	0.37	<0.001 *
**EMG rest (µV)**									
TA-R	2.23 ± 1.62	2.05 ± 1.09	0.17 ± 1.02	0.46	2.24 ± 0.94	2.21 ± 1.00	0.02 ± 0.52	0.80	0.74
TA-L	1.97 ± 1.14	1.69 ± 1.49	0.27 ± 0.79	0.19	1.70 ± 0.95	2.04 ± 1.84	−0.33 ± 1.36	0.28	0.04 *
MM-R	1.61 ± 0.74	1.44 ± 0.53	0.17 ± 0.64	0.09	1.33 ± 0.63	1.20 ± 0.59	0.12 ± 0.69	0.42	0.98
MM-L	1.43 ± 0.54	1.55 ± 0.77	−0.11 ± 0.70	0.50	1.50 ± 0.85	1.33 ± 0.46	0.16 ± 0.65	0.26	0.22
**EMG clench (µV)**									
TA-R	108.94 ± 60.40	102.84 ± 48.63	6.48 ± 43.21	0.52	53.05 ± 19.72	48.54 ± 18.94	4.51 ± 9.58	0.55	0.95
TA-L	122.03 ± 65.91	125.71 ± 63.93	−0.97 ± 49.97	0.93	48.59 ± 20.80	46.41 ± 19.69	2.18 ± 10.69	0.37	0.65
MM-R	131.09 ± 85.84	118.28 ± 70.22	10.79 ± 77.35	0.55	38.91 ± 28.91	34.30 ± 21.46	4.61 ± 10.57	0.06	0.53
MM-L	115.80 ± 60.47	110.74 ± 71.96	−3.06 ± 78.13	0.78	34.90 ± 22.33	33.12 ± 22.40	0.60 ± 11.26	0.25	0.46

* Statistically significant between-group difference. TA-L significance did not remain after correction for multiple comparisons.

## Data Availability

The data presented in this study are available from the corresponding author upon reasonable request. The data are not publicly available due to privacy and ethical restrictions involving human participants.
